# Disease activity and response to therapy monitored by [^18^F]FDG PET/CT using volume-based indices in IgG4-related disease

**DOI:** 10.1186/s13550-020-00743-w

**Published:** 2020-12-09

**Authors:** Katsuya Mitamura, Hanae Arai-Okuda, Yuka Yamamoto, Takashi Norikane, Yasukage Takami, Kengo Fujimoto, Risa Wakiya, Hiroki Ozaki, Hiroaki Dobashi, Yoshihiro Nishiyama

**Affiliations:** 1 Department of Radiology, Faculty of Medicine, Kagawa University, 1750-1 Ikenobe, Miki-cho, Kita-gun, Kagawa 761-0793 Japan; 2 Division of Hematology, Rheumatology and Respiratory Medicine, Department of Internal Medicine, Faculty of Medicine, Kagawa University, Kita-gun, Kagawa Japan

**Keywords:** [^18^F]FDG, PET, IgG4-related disease

## Abstract

**Purpose:**

The efficiency of [^18^F]FDG PET/CT using volume-based indices was evaluated to assess the disease activity and response to therapy in patients with immunoglobulin G4-related disease (IgG4-RD).

**Methods:**

A total of 17 patients with IgG4-RD were examined with [^18^F]FDG PET/CT before and during treatment. The lesion boundary was determined using a fixed threshold of standardized uptake value (SUV) ≥ 2.5. The highest maximum SUV (SUVmax) among all affected lesions was calculated for individual patients. We summed metabolic tumor volume (MTV) and total lesion glycolysis (TLG) of each affected lesion to generate a total MTV and total TLG. PET results were compared with those of serum IgG4 and soluble interleukin-2 receptor (sIL-2R) levels.

**Results:**

The mean number of involved organs per patient was 3.8 as determined by [^18^F]FDG uptake. The number of involved organs, total MTV and total TLG were significantly correlated with IgG4 (*P* = 0.046, < 0.001, < 0.001, respectively) and sIL-2R (*P* < 0.001, = 0.031, 0.031, respectively). According to the clinical assessments for therapy response, all patients were classified as improved. The SUVmax, total MTV, and total TLG during therapy were all significantly lower than those before therapy (all *P* < 0.001).

**Conclusion:**

[^18^F]FDG PET/CT is valuable for assessing the extent of multi-organ involvement before therapy and monitoring subsequent therapy in patients with IgG4-RD. [^18^F]FDG PET/CT using volumetric indices correlated with serum IgG4 and sIL-2R levels.

## Introduction

Immunoglobulin G4-related disease (IgG4-RD) is a systemic fibro-inflammatory condition characterized by diffuse or focal organ enlargement containing mass-forming lesions with copious IgG4-positive plasma cells [1, 2]. Since it is usually systemic with involvement of 2 or more organs, it often mimics sarcoidosis or malignancies with multiple metastases [2]. Clinically, more than half of the patients have elevated serum IgG4 levels, and the initial response to corticosteroid-based treatment is usually good, although relapses are frequent [2]. Despite the various sets of diagnostic criteria that have been proposed [3], the clinical diagnosis of IgG4-RD remains difficult because most patients present with only mild symptoms and the clinical spectrum is extremely diverse. This makes it important to further establish the role of various noninvasive imaging techniques in the differential diagnosis of suspected IgG4-RD.

2-deoxy-2-[^18^F]fluoro-D-glucose ([^18^F]FDG) accumulates not only in malignant lesions but also in inflammatory ones where glucose-consuming inflammatory cells infiltrate [4, 5]. [^18^F]FDG positron emission tomography/computed tomography (PET/CT) is useful in patients with IgG4-RD because it delineates the disease distribution in the whole body [6–13]. However, only a few studies have focused on the response to therapy in this population as determined by [^18^F]FDG PET/CT [7, 9, 11]. The most commonly used PET semiquantitative index is a standardized uptake value (SUV). Because of its simplicity, the maximum SUV (SUVmax) within the lesion is applied mostly to express the intensity of [^18^F]FDG uptake in the lesion clinically. Recently, volume-based indices such as the metabolic tumor volume (MTV) and total lesion glycolysis (TLG) are also useful in assessing disease activity [14]. Their advantage is to reflect the activity of the glucose metabolism in the entire lesion, whereas the SUVmax reflects that of only a single voxel.

This prompted us to evaluate the efficiency of [^18^F]FDG PET/CT using volume-based indices to assess the disease activity compared with serum inflammatory biomarkers and to monitor the response to therapy, in patients with IgG4-RD.

## Materials and methods

### Patients

This retrospective study was approved by the Kagawa University institutional review board with the requirement for obtaining informed consent waived. From September 2010 to July 2020, 17 patients (13 males, 4 females; mean age, 67.8 years; age range, 41–87 years) with IgG4-RD who underwent [^18^F]FDG PET/CT studies both before and during therapy were included in the study. All patients fulfilled the 2019 American College of Rheumatology/European League Against Rheumatism (2019 ACR/EULAR) Classification Criteria for IgG4-Related Disease [3]. Twelve patients had biopsy proven disease (biopsy site; 5 salivary glands, 2 cervical lymph nodes, 3 pancreas, 1 liver, 1 skin) and the mean total points of 2019 ACR/EULAR classification criteria per patient was 33 (median 30, range 20–53). Three of them had recurrent disease for which corticosteroid treatment had been administered within 6 months prior to the initial [^18^F]FDG PET/CT study. After the initial [^18^F]FDG PET/CT study, all patients received corticosteroid-based therapy. Serum levels of inflammatory biomarkers including IgG4 and soluble IL-2 receptor (sIL-2R) were performed within one month before or after the [^18^F]FDG PET/CT study.

### PET/CT imaging

[^18^F]FDG was produced using an automated synthesis system with HM-18 cyclotron (QUPID; Sumitomo Heavy Industries Ltd, Tokyo, Japan).

All acquisitions were performed using a hybrid PET/CT scanner (Biograph mCT, Siemens Medical Solutions USA Inc., Knoxville, TN, USA), which has an axial field of view of 21.6 cm and a 64-slice multi-detector CT scanner. Patients were instructed to fast for more than 5 h before [^18^F]FDG administration. A normal glucose level in the peripheral blood was confirmed before injecting the [^18^F]FDG. PET emission scanning (2 min per bed position) was performed 90 min after intravenous injection of [^18^F]FDG (5 MBq/kg) from the skull to upper-thigh, and co-registered with a non-contrast-enhanced CT of the same region. The PET data were reconstructed with a baseline ordered-subset expectation maximization algorithm, incorporating correction with point-spread function and time-of-flight model (2 iterations, 21 subsets).

### PET/CT data analysis

[^18^F]FDG PET/CT images were first visually assessed by two board-certified nuclear medicine physicians independently. Any difference of opinion was resolved by consensus. Visually, abnormal [^18^F]FDG uptake seen in locations unaccounted for by the normal biodistribution of [^18^F]FDG were interpreted as lesions. The hilar/mediastinal inflammatory lymph nodes are commonly seen and nonspecific on [^18^F]FDG PET/CT scan. Therefore, interpreting hilar/mediastinal lymph nodes were excluded, unless there was other supporting data. Next, a board-certified nuclear medicine physician performed the semiquantitative analyses. The SUV was calculated using the following formula: SUV = *c*_dc_/(*d*_i_/*w*), where *c*_dc_ is the decay-corrected tracer tissue concentration (Bq/g); *d*_i_ is the injected dose (Bq); and w is the patient’s body weight (g). The lesion boundary was determined using a fixed threshold of SUV ≥ 2.5 based on a previous report [10]. In semiquantitative analysis, the urinary system was excluded because we could not completely exclude the influence of physiological excretion in the urine. The lesion volume was calculated as the MTV. The TLG was calculated as MTV × mean SUV within the same region. The highest SUVmax among all affected lesions was also evaluated for each patient. If there was no abnormal uptake during therapy, SUVmax of the previously involved organs on the basis of before therapy [^18^F]FDG PET/CT scan were measured. The percent change in SUVmax was calculated from before therapy (before) to during therapy (during) as follows: (during SUVmax – before SUVmax) × 100/ before SUVmax. The percent change in total MTV, total TLG, IgG4, and sIL-2R were calculated by the same manner. For the PET index calculation, we used Metavol, an open-source software tool developed for the efficient measurement of tumor volume on PET/CT [15]. For patient-based assessment, we summed the MTV and TLG of each affected lesion to generate a total MTV and total TLG.

### Clinical assessments of therapy response

The course of disease activity was evaluated according to the clinical criteria, which included improvement in patients’ symptoms, resolution or amelioration of physical findings, serial testing of serum inflammatory biomarkers, improvement on imaging studies, and ability to taper corticosteroid successfully [16]. Patients under therapy were then classified as improved, unchanged, or worsened.

### Statistical analysis

All statistical analyses were performed using a software package (SPSS Statistics, version 26; IBM). Data were analyzed for statistical significance using Wilcoxon signed-rank test and Spearman’s correlation coefficient. Differences were considered statistically significant at *P* values less than 0.05.

## Results

### [^18^F]FDG uptake before therapy and serum inflammatory biomarkers

[^18^F]FDG uptake was visually abnormal in at least one organ before therapy in all patients. Table 1 shows the results of IgG4-RD organs with [^18^F]FDG uptake. The most common involved organ was the salivary gland, followed by the cervical lymph nodes. The mean number of involved organs with [^18^F]FDG uptake per patient was 3.8 (median 3, range 1–6) and multiple organ involvement was found in 94% (16/17) patients. Visually, [^18^F]FDG uptake for the organs that exist bilaterally showed an almost symmetrical distribution. There was no significant laterality of SUVmax, MTV, and TLG for abnormal uptake (*P* = 0.687, 0.528, 0.658, respectively).

**Table 1:** Organs with [^18^F]FDG uptake before therapy in patients with IgG4-RD

Organs with abnormal [^18^F]FDG uptake	*n*
Lacrimal gland	3 (18%)
Salivary gland	13 (76%)
Cervical lymph nodes	10 (59%)
Abdominal lymph nodes	8 (47%)
Lung	1 (6%)
Pancreas	8 (47%)
Bile duct	1 (6%)
Kidney	4 (24%)
Aorta	7 (41%)
Retroperitoneum	3 (18%)
Prostate	8 (47%)
Spermatic cord	1 (6%)
Skin	1 (6%)

Table 2 shows the results of correlation of [^18^F]FDG PET findings with serum inflammatory biomarkers. Four patients lacked sIL-2R data. There were no significant correlations between SUVmax and any of the serum biomarkers. The number of involved organs, total MTV and total TLG showed a significant correlation with IgG4 (*P* = 0.046, < 0.001, < 0.001, respectively) and sIL-2R (*P* < 0.001, = 0.031, 0.031, respectively).

**Table 2:** Correlation of [^18^F]FDG PET indices before therapy with serum inflammatory biomarkers in patients with IgG4-RD

[^18^F]FDG PET	Serum inflammatory biomarkers			
	IgG4 (*n* = 17)		sIL-2R (*n* = 13)	
	*ρ*	*P*	*ρ*	*P*
Number of involved organs	0.490	0.046	0.873	< 0.001
SUVmax	0.455	0.067	0.467	0.108
Total MTV	0.879	< 0.001	0.599	0.031
Total TLG	0.873	< 0.001	0.599	0.031

sIL-2R = soluble IL-2 receptor, SUVmax = maximum standardized uptake value, MTV = metabolic tumor volume, TLG = total lesion glycolysis

### Changes of [^18^F]FDG uptake and serum inflammatory biomarkers during therapy, and clinical assessments

Fifteen patients had received corticosteroid treatment, and the remaining two immunosuppressive agents as well. The median delay between initiation of corticosteroid-based therapy and [^18^F]FDG PET/CT scan during therapy was 44 days (range 7–1252 days). During therapy, the mean number of involved organs according to [^18^F]FDG uptake per patient was 1 (median 1, range 0–5) and complete disappearance of [^18^F]FDG uptake was observed in 8 of 17 (47%). The mean number of involved organs showing [^18^F]FDG uptake during therapy was significantly lower than that before therapy (*P* < 0.001).

**Fig. 1:**
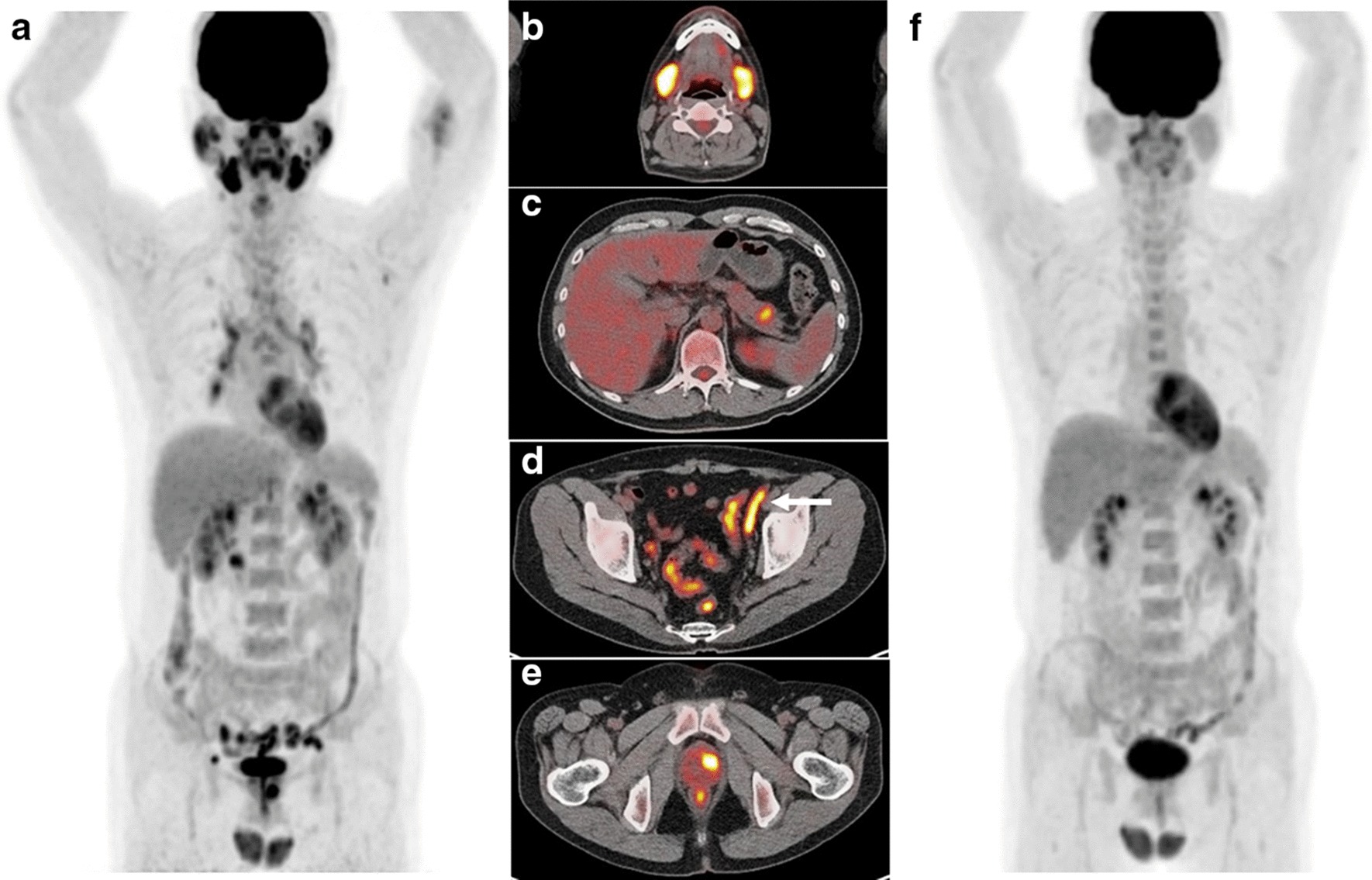
Pretreatment [^18^F]FDG PET maximum intensity projection (MIP) image (**a**) and transverse fused PET/CT images **b**–**e** of a 45-year old male with IgG4-related disease shows multiple organ involvement, including of salivary glands, cervical lymph nodes, pancreas (head and tail), spermatic cord (arrow), and prostate. Thirty-six days after initiation of corticosteroid therapy, [^18^F]FDG PET MIP image **f** shows complete disappearance of the abnormal [^18^F]FDG uptake

According to the clinical assessments of the therapeutic response, all patients were classified as improved. Symptomatically, 13 patients with salivary gland swelling and 2 patients with urinary tract obstruction reported some improvement in symptoms. Radiologically, 3 patients with enlargement of lacrimal gland, 13 patients with enlargement of salivary gland, 8 patients with enlargement of pancreas, and 13 patients with lymphadenopathy reported some improvement on imaging studies. Serologically, serum IgG4 levels decreased in 100% (17/17) and with significance (*P* < 0.001). Although sIL-2R both before and during therapy were available in 6 patients, serum sIL-2R levels decreased in 83% (5/6) and with less significance. The SUVmax, total MTV and total TLG values were decreased relative to before treatment to during therapy in all patients. Table 3 shows the results of the [^18^F]FDG PET findings before and during therapy. The values of SUVmax, total MTV, and total TLG during therapy were significantly lower than those before therapy, respectively (all *P* < 0.001). Between percent change of [^18^F]FDG PET indices and serum biomarkers, no significant correlations were found in any of SUVmax and IgG4 (*P* = 0.970), SUVmax and IL-2R (*P* = 0.957), total MTV and IgG4 (*P* = 0.840), total MTV and IL-2R (*P* = 0.389), total TLG and IgG4 (*P* = 0.634), or total TLG and IL-2R (*P* = 0.389). Representative [^18^F]FDG PET/CT images before and during therapy are shown in Fig. 1.

**Table 3:** [^18^F]FDG PET indices before and during therapy in patients with IgG4-RD

[^18^F]FDG PET	Before therapy		During therapy		*P* value
	Mean	SD	Mean	SD	
SUVmax	7.49	2.35	3.55	3.09	< 0.001
Total MTV	131.0	133.7	13.4	25.2	< 0.001
Total TLG	456.1	470.3	44.7	86.5	< 0.001

SUVmax = maximum standardized uptake value, MTV = metabolic tumor volume, TLG = total lesion glycolysis

## Discussion

The present findings documented the value of [^18^F]FDG PET in assessing multi-organ involvement as well as the relatedness of volumetric PET analysis and IgG4 and sIL-2R levels, in patients with IgG4-RD. [^18^F]FDG PET was also feasible for assessing therapy monitoring.

Assessment of disease activity is important because early intervention is effective in preventing irreversible organ damage in IgG4-RD [17]. And because it is a multiple organ systemic disease, evaluation of the disease distribution in the whole-body is important. [^18^F]FDG PET/CT can assess the distribution of multiple involved regions in the entire body in one scan. A recent study found multiple organ involvement in 97.1% patients [11], consistent with the present result. Yabusaki et al. assessed the vascular involvement in IgG4-RD and found that involved vascular showed more than twice the [^18^F]FDG uptake as compared to the venous blood pool [13]. Ebbo and colleagues demonstrated that [^18^F]FDG PET using qualitative analysis was more sensitive than conventional imaging in detecting active lesions [9]. To date, few studies have evaluated volumetric PET analysis in patients with IgG4-RD [10, 11]. In the present study, number of involved organs, total MTV and total TLG were significantly correlated with serum biomarkers, whereas SUVmax was not. SUVmax is the most commonly used quantitative PET parameter, but it reflects only a single voxel, and this limitation needs to be kept in mind.

Tabata et al. concluded that measurement of serial serum IgG4 levels is helpful to assess the disease activity of IgG4-RD [18]. In the preset study, serum IgG4 levels also significantly correlated with total MTV and total TLG. In contrast, two other previous studies did not find TLG to correlate with serum IgG4 levels [10, 11]. In these previous studies, the diagnoses of IgG4-RD were based on the criteria of Umehara et al. [19], whereas in the present study the diagnosis of IgG4-RD was based on the 2019 ACR/EULAR classification criteria. In addition, the method of calculating TLG in Zhang et al.’s study was not specified [11]. On the other hand, in Nakatsuka et al.’s study, serum sIL-2R levels significantly correlated with total TLG [10]. This finding is in line with that in the present study. sIL-2R is considered to have an important role in lymphocyte activation in patients with IgG4-RD [20]. To date, there is still very limited experience of [^18^F]FDG PET as related to serum biomarkers in patients with IgG4-RD. Further studies will be needed to better clarify the correlation between [^18^F]FDG PET findings and disease biology in this disease.

Regarding the treatment response, in the present study, the mean number of involved organs with [^18^F]FDG uptake during treatment was significantly lower than that before therapy and 47% patients had a complete disappearance of abnormal [^18^F]FDG uptake after treatment. Two previous studies by Ebbo et al. and Zhang et al. demonstrated a complete metabolic remission in 45% and 72%, respectively [9, 11]. The interval between initiation of therapy and follow-up [^18^F]FDG PET/CT scan was very different in the present and these previous studies. Ebbo et al. reported a good relation between qualitative [^18^F]FDG PET results and therapeutic response in 11 patients with IgG4-RD [9]. Nakatsuka et al. evaluated only 6 patients in whom serial changes in total TLG were followed although only 3 patients had received therapy [10]. Four patients who exhibited decreases in total TLG showed clinical improvement, whereas two who exhibited increases in total TLG showed clinical deterioration [10]. Meanwhile, SUVmax was not necessarily associated with the clinical course [10]. The volumetric [^18^F]FDG PET parameters, rather than SUVmax, might be useful to assess the therapeutic response.

Limitations of the present study include small sample size and retrospective design. Pathological analysis was not always obtained for all suspected lesions. Intervals between start of treatment and during therapy [^18^F]FDG PET/CT scan were not uniform. Although IgG4-RD not infrequently involves the urinary tract, it was excluded for semiquantitative analysis in the present study because of interference by physiological excretion in the urine.

[^18^F]FDG PET/CT offers the additional benefit of malignancy detection in this population, which is of particular importance given the increased prevalence of malignant tumors that has been reported in it [21]. Further additional large prospective studies are needed to confirm our results and to elucidate their potential clinical value in patients with IgG4-RD.

## Conclusion

These preliminary results suggested that [^18^F]FDG PET/CT is useful for assessing multi-organ involvement before therapy and for therapy monitoring in patients with IgG4-RD. [^18^F]FDG PET/CT using volumetric indices were related with serum IgG4 and sIL-2R levels.

## Data Availability

All results are provided in the manuscript.
